# Predictors of Weaning Success in Patients on Prolonged Mechanical Ventilation: A Retrospective Cohort Study

**DOI:** 10.3390/jcm14134427

**Published:** 2025-06-22

**Authors:** Bartal Amir, Ofri Mai, Turgeman Shira, Peles Ido, Paran Nave, Bartal Carmi

**Affiliations:** 1 Internal Medicine Department, Soroka University Medical Center, Ben Gurion University of Negev, Beer Sheva P.O. Box 151, Israel; amirbt8@gmail.com (B.A.); maiof@post.bgu.ac.il (O.M.); shira259@gmail.com (T.S.); idopeles17@gmail.com (P.I.); navepa@clalit.org.il (P.N.); 2 Clinical Research Center, Soroka University Medical Center, Beer Sheva P.O. Box 151, Israel

**Keywords:** mechanical ventilation, weaning, hypoalbuminemia, Sequential Organ Failure Assessment score, critical care, tracheostomy, long-term ventilation

## Abstract

**Background/Objectives:** Weaning failure remains a major challenge in patients requiring prolonged mechanical ventilation. This study aimed to describe outcomes in patients ventilated for >14 days and identify specific predictors of weaning success. **Methods:** A retrospective analysis of 88 patients from the Soroka University Medical Center database was conducted. Outcomes in the successful weaning (SW) group were compared to those in the failed weaning (FW) group. Predictors of weaning success were analyzed using multivariate logistic regression. **Results:** Forty patients (45%) were successfully weaned and discharged to rehabilitation or home. In-hospital mortality was 28%, with deaths occurring exclusively in the FW group (*p* < 0.001). One-month and one-year post-discharge all-cause mortality rates were 11% and 28%, respectively, with no group differences. Hypoalbuminemia and the Sequential Organ Failure Assessment (SOFA) score at admission significantly predicted weaning failure (odds ratio: 5.71 and 0.54, respectively). Demographics, comorbidities, ventilation indications, admission data, and diuretic use were not predictive. **Conclusions:** Hypoalbuminemia and the SOFA score at admission were key predictors of weaning success in patients ventilated for more than 2 weeks. Age and comorbidities were not significant. Prospective studies on albumin supplementation and high-protein diets are warranted to assess their impact on weaning outcomes.

## 1. Introduction

Prolonged mechanical ventilation is a growing challenge in critical care, contributing significantly to morbidity, mortality, and healthcare resource utilization. Weaning from mechanical ventilation is defined as the first attempt at separating a patient from a ventilator, regardless of the modality used, immediately after the acute condition necessitating invasive mechanical ventilation has been resolved [1,2]. Delays in the weaning process have been associated with increased complication rates, prolonged hospital stays, and reduced intensive care unit (ICU) survival rates [3,4,5].

In 2017, a new definition and classification system, known as the Weaning Outcome According to a New Definition (WIND), was introduced to better categorize weaning outcomes and allow for comparisons across different ICUs. This system included four groups: (class 1) short weaning (>24 h); (class 2) difficult weaning (between 1 day and 1 week); (class 3a) successful weaning after >1 week; and (class 3b) never experienced any separation attempt [6,7,8]. However, the WIND classification criteria do not account for patients transferred to respiratory weaning centers after unsuccessful ICU weaning attempts (class 3b). Notably, patients who require prolonged invasive mechanical ventilation for several weeks may still eventually be successfully weaned [9,10,11,12].

Bolles summarized several factors that predict weaning failure, including respiratory effort, cardiac function, nutritional status, and neurological condition [2]. Additionally, Sellares et al. observed that chronic obstructive pulmonary disease (COPD) could help predict longer hospital stays and lower survival rates in patients with prolonged ventilation [13]. Upadia et al. demonstrated that fluid balance is an important factor in successful weaning, even on the first day of mechanical ventilation [14]. Several studies have indicated that maintaining a negative fluid balance through diuretic use improves early weaning outcomes in critically ill patients [15,16]. However, there remains considerable debate regarding which clinical factors most reliably predict weaning outcomes in patients with prolonged mechanical ventilation. Moreover, the efficacy and safety of diuretic treatment during the stabilization phase of critical illness, usually >1 week after the initiation of mechanical ventilation, have not been thoroughly investigated [17].

Specific data regarding predictors of weaning failure in patients ventilated for >1 week are lacking. At our ICU, it is common practice to continue weaning efforts in stable patients with mainly one-organ failure (respiratory failure) after 2 weeks of continuous mechanical ventilation before transferring them to a pulmonary rehabilitation unit. Therefore, this study primarily aimed to identify predictors of weaning success by comparing successful and failed weaning groups. The secondary aim was to describe the clinical outcomes of patients with prolonged mechanical ventilation.

Our hypothesis was that some predictors of successful weaning from prolonged mechanical ventilation may be associated with the severity of illness upon admission, including the Sequential Organ Failure Assessment (SOFA) score, age, nutritional status, and initial serum albumin level in particular, and that these factors continue to influence the likelihood of successful weaning even after stabilization of the critical condition that necessitated intubation. We also hypothesized that diuretic therapy, once the critical condition has stabilized, may contribute to successful weaning. Identifying such predictors could allow for earlier interventions by the healthcare team during the initial phases of mechanical ventilation, potentially improving the chances of weaning in patients undergoing prolonged ventilation.

## 2. Materials and Methods

### 2.1. Study Design and Setting

This retrospective cohort study included patients on continuous, prolonged ventilation (≥14 days) who had at least one weaning attempt at Soroka University Medical Center (SUMC) between 2015 and 2024. This study was performed using the computerized database of SUMC, a tertiary 1191-bed single medical center serving 1.2 million residents in Southern Israel.

### 2.2. Study Population

This study included patients aged ≥18 years who were hospitalized at a respiratory rehabilitation unit inside the internal medicine ward and had been continuously ventilated for >14 days, having failed at least one extubation trial in the ICU or Internal Medicine Department. Patients were excluded if they had been on ventilation for less than 14 days, were designated as not resuscitating, had severe dementia (with their pre-intubation functional status indicating a lack of decision-making capacity), were bedridden, had end-stage lung or liver disease, had a life expectancy of six months or less, or had active malignancies. The study population was divided into two groups: the successful weaning (SW) group was compared with the failed weaning (FW) group.

### 2.3. Weaning Protocol and Definition of Weaning Success

All patients in the cohort were managed using our institutional uniform weaning protocol. Sedation was achieved using either propofol or midazolam. Patients admitted to our department were not treated with neuromuscular blocking agents. However, some patients who had been previously admitted to intensive care units did receive neuromuscular blockers during the first two weeks of their illness. The patients were exposed to a gradual decrease in pressure-support ventilation. Once an inspiratory pressure of 10–12 cm H_2_O was achieved, enabling the creation of a tidal volume of >350 cc and a respiratory rate of <28/min, we conducted the first spontaneous breathing test (SBT). The SBT protocol consisted of stepwise T-piece oxygen flow support: initially for 4 h, followed by a 12 h SBT and finally an overnight SBT before attempting an extubation trial. Complete successful extubation was defined as spontaneous breathing without mechanical ventilation for more than 48 consecutive hours, with or without noninvasive ventilation (with high-flow ventilation such as bilevel positive airway pressure (BiPAP) for less than 12 h per day). We did not adopt the criteria proposed by Virole et al. [18]. Dependence on BiPAP for more than 12 h per day was defined as a weaning failure.

This study was performed in accordance with the Declaration of Helsinki and approved by the Institutional Review Board of SUMC (protocol code 0164-23-SOR). The requirement for informed consent was waived, as all data were collected retrospectively and participants’ identities remained anonymous.

### 2.4. Study Outcomes

The potential predictors of successful weaning were explored in patients undergoing very prolonged mechanical ventilation. Further, we examined and compared the mortality at 1 month and 1 year, the weaning success rate, and the hospitalization length of stay between the two groups.

### 2.5. Data Sources

Computerized data including demographic characteristics, diagnoses from hospitalizations, chronic illnesses, laboratory data, intubation data, parameters, treatments administered before the extubation trial, and clinical outcomes were extracted from the SUMC database.

### 2.6. Statistical Analysis

We hypothesized that weaning failure is associated with the clinical background, administered medications, and ventilation properties. The sample size included 88 participants: 48 in the failed weaning group and 40 in the successful weaning group. This sample size was sufficient to detect effect sizes of 1.05 with α = 0.05 and statistical power exceeding 80%. Initially, a descriptive analysis was performed to summarize the central tendency and distribution of variables related to patient demographics, clinical backgrounds, laboratory findings, medications administered, and clinical outcomes. Univariate analysis was then conducted. Pearson’s chi-square test, Fisher’s exact test, *t*-test, and Wilcoxon rank-sum test were used to compare categorical, normally distributed, and non-normally distributed continuous variables, respectively. Subsequently, multivariate logistic regression analysis was performed to evaluate variation in inflation factors. Variables were included in the regression analysis based on domain expertise and univariate analysis. All statistical analyses were conducted using R software version 4.2.0.

## 3. Results

**Figure 1:**
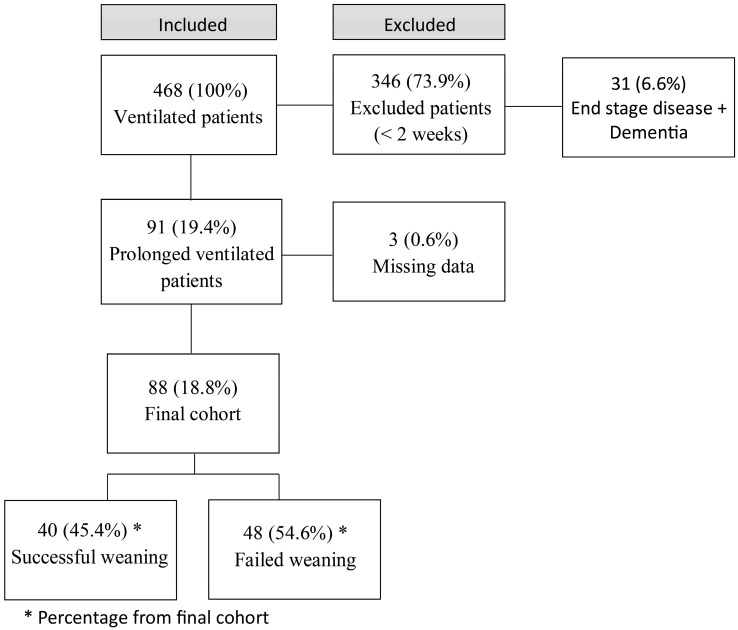
Study flow chart.

A total of 468 ventilated patients were included in the initial cohort; of these, 377 were excluded based on exclusion criteria, and 3 patients were further excluded due to missing data or transfer to another unit. A final cohort of 88 patients who required prolonged mechanical ventilation (defined as ventilation for >14 days) underwent weaning trials at the respiratory rehabilitation unit inside the Internal Medicine Department (Figure 1). The cohort was divided into two groups based on weaning outcomes (SW and FW groups, including 40 and 48 patients, respectively). Table 1 presents the baseline demographic characteristics and clinical backgrounds of both groups, while the main reasons for intubation among patients are summarized in Table S1. No significant differences were identified in sex, social status, background diseases, or reasons for intubation between the SW and FW groups (Table S1). However, patients in the SW group were younger [median age: 64 years (range: 57–76)] than those in the FW group [median age: 77 years (range: 67–82)] (*p* < 0.05). Hypertension was not a significant predictor of weaning failure (*p* = 0.07). Other comorbidities such as COPD, obesity, diabetes, and chronic ischemic heart disease did not affect outcomes.

Table 2 presents clinical parameters hypothesized to influence outcomes between the two groups. The SOFA score was significantly higher in the FW group than in the SW group (8.31 vs. 7.41, *p* = 0.011). The initial pressure for oxygen flow (i.e., PaO_2_/FiO_2_ ratio or P/F ratio) was significantly lower in the FW group than in the SW group (89 vs. 142, *p* = 0.008). No significant differences were noted in the duration of ventilatory support prior to weaning initiation (*p* = 0.12) or complications during mechanical ventilation, such as ICU-acquired polyneuropathy (*p* = 0.09) between the two groups.

Among laboratory parameters, a significant difference was observed in the serum albumin levels at admission between the two groups (*p* < 0.005). SW patients had higher albumin levels (mean: 2.78 g/dL (SD = 0.4)) than FW patients (mean: 2.36 g/dL (SD = 0.4)). Urea levels, but not creatinine levels, were significantly higher in the FW group than in the SW group (74 vs. 43 mg/dL, *p* = 0.008).

Table 3 summarizes the main outcomes of this study. Overall, 40 patients (45%) were successfully weaned and subsequently discharged to rehabilitation facilities or directly to their homes. Of these, 58% required no ventilatory support, 38% required continuous oxygen via nasal cannula, and 5% required noninvasive mechanical ventilation (BiPAP) or high-flow oxygen therapy. The mean hospitalization length of stay and duration of mechanical ventilation were 45 days (IQR: 32–59 days) and 31 days (IQR: 19–41 days), respectively, with no significant difference between the two groups. Most patients in both the groups underwent tracheostomy (78% in the SW group vs. 79% in the FW group). The duration of mechanical ventilation was inversely associated with weaning outcomes: FW patients required a median of 35 days of ventilation compared with 28 days required by SW patients (*p* = 0.04).

In-hospital mortality was significantly higher in the FW group (52%; 25 patients), while no deaths occurred in the SW group. However, 1-year post-discharge mortality was 25% in the FW group (77% if in-hospital deaths are included) versus 33% in the SW group, with no significant difference. Further, no significant differences were observed in secondary outcomes, including all-cause mortality within 1 month of discharge or hospitalization length of stay. Multivariate logistic regression analysis identified only the admission albumin level and SOFA score as significant predictors of weaning success (Table 4).

Post hoc power analysis of the “diuretics daily use” variable was conducted on the 64 patients that received diuretic agents. In the FW group, 37 patients received diuretics, with a mean and standard deviation of 71 ± 61 mg/day. In the SW group, 27 patients received diuretic agents, with a mean and standard deviation of 50 ± 34 mg/day. The minimal difference to detect was 37 mg/day, to achieve a power of 80%.

Proposed main conclusion: After adjusting for SOFA score, age, and urea level, higher albumin levels were associated with higher odds of successful weaning. Additionally, a higher SOFA score was significantly associated with lower odds of successful weaning.

These findings demonstrate that hypoalbuminemia and higher SOFA scores on admission are significant predictors of weaning failure among patients undergoing prolonged mechanical ventilation.

## 4. Discussion

This study was designed to identify predictors of successful weaning in patients with mechanical ventilation, and our findings revealed hypoalbuminemia and SOFA scores on admission as significant determinants. Prolonged weaning from mechanical ventilation has been associated with extreme consequences on patient health, life expectancy, and quality of life. Overall, one-quarter of critically ill patients require mechanical ventilation for >1 week. Moreover, approximately 10% of patients require ventilation 30 days after initiation [19]. This subgroup of patients accounts for approximately half of the resources used in the ICU. Therefore, many of these patients are subsequently transferred to specialized long-term care facilities designed for patients requiring extended periods of ventilation.

This study population included patients who were on ventilation for at least 2 weeks and subsequently failed to wean, classified into class 3b according to the WIND criteria [12,20]. Why did we choose to investigate this patient population? There were several reasons for this focus. First, this group comprises the primary workload of respiratory rehabilitation units, including our own hospital-based unit. According to the WIND classification criteria, class 3b has scarcely been studied, and there is limited scientific literature regarding its clinical outcomes, such as weaning success rates and long-term mortality. Second, most patients who cannot be weaned within 2 weeks and for whom successful weaning is not anticipated in the near future undergo tracheostomy. Following failed weaning attempts and tracheostomy, these patients are typically transferred from ICUs to long-term respiratory care units or weaning facilities. This practice is primarily due to the low probability of successful weaning and the limited added benefit of advanced ICU-level care compared to specialized respiratory rehabilitation centers. Third, patients who remain ventilated for >2 weeks, as opposed to those ventilated for just >1 week, typically achieve a degree of clinical stability. This stability is characterized by control of the primary disease, which initially led to respiratory failure and mechanical ventilation. At this stage, ICU capabilities no longer meaningfully contribute to patient improvement.

The weaning success rate in our cohort was 45% at 1 year, which is much lower than the 64% reported by Windisch in Germany [21]. However, 20% of the patients in the Windisch study who were successfully weaned remained dependent on noninvasive mechanical ventilation. In contrast, only 5% of the patients in our study required noninvasive ventilation (BiPAP), which could explain the different results. In the WEAN-SAFE trial conducted by Pham et al., patients who failed to wean from mechanical ventilation had a 1-year mortality of approximately 75% [19], which is similar to the 77% in our study. However, 52% of the deaths in our study occurred during ICU hospitalization, whereas 25% of our patients died during the first year after ICU discharge. Meanwhile, the mortality at 1 year in successfully weaned patients was higher in our cohort (33%) than the 15–20% reported in the literature [20,21]. Further, no significant difference was noted in the all-cause mortality at 1 month and 1 year between the two groups in our study. Herein, the mortality in both groups was higher than that in the Windisch cohort (14.5%) and the WEAN- SAFE trial (25%). This discrepancy may be attributed to the different inclusion criteria: in both multi-center trials, patients were included after >1 week of mechanical ventilation and not after >2 weeks, as in our study, giving more time to achieve a higher weaning success rate. Another explanation for this difference could be the higher severity of illness in our patients (evidenced by factors such as the higher SOFA scores upon admission and fewer patients with acute respiratory distress syndrome).

The probability of weaning failure in patients on ventilation for >14 days after an extended weaning period remains unclear, as it is based on very few studies [20,21]. Therefore, we aimed to identify predictors of successful or unsuccessful weaning in patients undergoing prolonged mechanical ventilation. In 2002, Esteban demonstrated that specific underlying conditions, such as severe sepsis and multiorgan failure, particularly acute respiratory distress syndrome, necessitate prolonged mechanical ventilation [2]. In a 2007 review, Boles identified several predictors of successful weaning from mechanical ventilation [2]. These predictors include respiratory diseases, neuromuscular disorders, cardiac function, nutritional status (low body mass), psychological state (presence of delirium), diaphragm function, hyperglycemia, anemia, metabolic acidosis, renal failure, and electrolyte imbalances [18,22,23,24]. Pilcher et al. showed that the probability of successful weaning from extended mechanical ventilation was lower in patients with neuromuscular disorders or compromised chest wall integrity than in those with COPD. However, an inverse relationship was identified regarding mortality, with significantly higher mortality observed in individuals with prolonged ventilation due to COPD than in those with neuromuscular disorders [3]. In the WEAN-SAFE trial in 2023, Pham et al. showed that advanced age was the primary cause of in-hospital mortality among patients mechanically ventilated in the ICU for >1 week. Additionally, the trial identified other major factors contributing to weaning failure in this patient group, such as the duration of mechanical ventilation in the ICU, underweight status, and the presence of pre-existing neuromuscular disorders [19].

In a 2019 meta-analysis by Ghauri et al., COPD and renal insufficiency were identified as predictors of prolonged ventilation. Furthermore, similar findings were observed in patients who received mechanical ventilation after neurovascular events and endovascular treatment [22,23,25].

In our study, the predictors of weaning failure included advanced age, duration of ventilation in the ICU and before extubation, SOFA score, renal insufficiency, and hypoalbuminemia at admission, consistent with other previously reported findings [2,19,21,22]. However, logistic regression analysis revealed that hypoalbuminemia and SOFA score on admission were the only independent predictors of prolonged weaning success (odds ratio: 5.71 (*p* = 0.008) and 0.54 (*p* = 0.012), respectively). Although many previous studies have reported components of the SOFA score to be predictive of weaning success, our findings reinforce the idea that baseline clinical conditions at the time of admission remain crucial in determining weaning outcomes for patients receiving mechanical ventilation for >2 weeks. However, when performing logistic regression analysis for respiratory components such as hypoxia on admission (P/F score) and baseline renal function on admission (Table S2), none of these components emerged as solitary predictors with statistical significance. Our working hypothesis was that age and diuretic treatment before and during weaning efforts are relevant in the prognosis of weaning from mechanical ventilation; however, the logistic regression and post hoc analysis did not support this, possibly due to the limited sample size. Although we examined the cumulative dose of diuretics used in the treatment, we could not confirm our initial assumption, not only due to the sample size but also due to timing issues.

Hypoalbuminemia has previously been recognized as a pivotal predictor of mortality in patients with shock treated with extracorporeal membrane oxygenation therapy [26]. Nutritional status is a typical but well-known and crucial factor that influences weaning from mechanical ventilation. Furthermore, low body mass has been associated with weaning failure, which is consistent with the observation of hypoalbuminemia in our study. Therefore, hypoalbuminemia is the most significant predictor of weaning failure from mechanical ventilation, which may result from a multifactorial pathophysiology [27].

1. Increased risk of infections: Hypoalbuminemia increases the risk of infections, sepsis, and multiorgan failure, particularly increasing the likelihood of ventilator-associated pneumonia. Conde’s 2008 study [28] identified hypoalbuminemia as a predictor of postoperative pulmonary infections;
2. Altered pharmacokinetics and pharmacodynamics: The effectiveness of targeted antibiotic therapy is compromised by hypoalbuminemia, which affects treatment efficacy [20];
3. Negative protein balance: Hypoalbuminemia leads to muscle breakdown and respiratory muscle weakening. Some studies have shown that inspiratory muscle training increases inspiratory muscle strength in patients being weaned from mechanical ventilation [18,29];
4. Increased thromboembolic events: Hypoalbuminemia increases the risk of thromboembolic events, including large arterial and venous events such as deep vein thrombosis and pulmonary embolism;
5. Indication of poor nutritional status: Hypoalbuminemia signifies poor caloric balance and malnutrition, often due to chronic illness;
6. Critical for wound healing: Albumin is essential for wound healing. The presence of pressure ulcers in patients with hypoalbuminemia reduces the healing potential and worsens the prognosis.

Therefore, a prospective follow-up study is necessary to assess whether active treatment with albumin, either through jejunostomy feeding or daily intravenous administration, can improve weaning success.

This study has some limitations. This was a single-center, retrospective study; therefore, not all potential predictors of successful weaning from prolonged mechanical ventilation were examined. For example, diaphragmatic function is typically recognized as a significant factor in weaning success, lean body mass on admission, and left/right heart function; however, we could not obtain comprehensive data on these parameters in all ventilated patients.

Nevertheless, this study has some strengths. The weaning process was standardized, as it was conducted at the same center by the same physicians using consistent protocols. Additionally, our study cohort was relatively large and particularly focused on patients who had been on ventilation for >14 days, compared with other studies where prolonged ventilation was only one of the several subgroups examined. The findings of this study have both therapeutic and educational implications. The standard treatment aiming to improve hypoalbuminemia usually necessitates enteral feeding via a nasogastric or post-pyloric tube to achieve the nutritional targets. Concurrently, physiotherapists work to enhance the function of the inspiratory muscles in an effort to facilitate successful weaning. Nevertheless, such efforts are unlikely to succeed without concurrent nutritional correction. The main results of this study suggest the need for early intervention by ICU clinicians in ventilated patients who have not been weaned within the first 24 h through the incorporation of treatment components aimed at improving predictors associated with prolonged weaning failure (lasting more than one week). These interventions may include treatments focused on improving the function of organ systems relevant to the SOFA score and aggressive nutritional support such as intravenous albumin administration to maintain albumin levels above 3 gl/L, or the use of total parenteral nutrition (TPN) or partial peripheral parenteral nutrition (PPN) in parallel with oral feeding. This could serve as a proactive strategy for improving respiratory muscle performance within a short time frame and thereby significantly enhance weaning outcomes.

There is no doubt that a prospective, multicenter study is required to assess additional predictors that were not examined in the current research.

## 5. Conclusions

This study demonstrated that hypoalbuminemia and SOFA scores on admission were the primary predictors of weaning success in patients on ventilation for >2 weeks. However, the importance of predictors such as age and background diseases was not ratified in this population. Therefore, our study has significant therapeutic implications, as daily albumin supplementation and a high-protein diet could be studied to determine whether they enhance weaning rates in a prospective study. These findings may contribute to a better understanding of factors influencing prolonged mechanical ventilation outcomes and inform clinical decision-making in ICU and rehabilitation settings.

## Figures and Tables

**Table 1:** Baseline demographic and clinical characteristics upon admission.

	Overall, N = 881	Failed Weaning Group (N = 48) ^1^	Successful Weaning Group (N = 40) ^1^	*p*-Value ^2^
Age (years)	73 (63, 80)	77 (67, 82)	64 (57, 76)	**0.002**
Sex—Male	44 (50%)	24 (50%)	20 (50%)	>0.9
Weaning attempts	3.00 (1.00, 3.50)	3.00 (2.00, 4.00)	2.00 (1.00, 3.00)	**0.026**
Hypertension	51 (58%)	32 (67%)	19 (48%)	0.070
Dyslipidemia	43 (49%)	24 (50%)	19 (48%)	0.8
Ischemic heart disease	34 (39%)	22 (46%)	12 (30%)	0.13
Diabetes mellitus	32 (36%)	18 (38%)	14 (35%)	0.8
COPD	23 (26%)	14 (29%)	9 (23%)	0.5
Obesity	22 (25%)	15 (31%)	7 (18%)	0.14
CKD	13 (15%)	7 (15%)	6 (15%)	>0.9
Malignancy	13 (15%)	9 (19%)	4 (10%)	0.08
Pulmonary hypertension	9 (10%)	6 (13%)	3 (7.5%)	0.5
Thyroid dysfunction	7 (8.0%)	3 (6.3%)	4 (10%)	0.7
OSA	5 (5.7%)	2 (4.2%)	3 (7.5%)	0.7
Dementia	5 (5.7%)	4 (8.3%)	1 (2.5%)	0.4
Smoking	17 (19%)	7 (15%)	10 (25%)	0.2
Alcohol	9 (10%)	5 (10%)	4 (10%)	>0.9
Drug use	6 (6.8%)	2 (4.2%)	4 (10%)	0.4
Chronic steroid use	1 (1.1%)	0 (0%)	1 (2.5%)	0.5

^1^ Median (IQR); n (%); ^2^ Wilcoxon rank-sum exact test; Pearson’s Chi-square test; Wilcoxon rank-sum test; Fisher’s exact test COPD, chronic obstructive pulmonary disease; CKD, chronic kidney disease; OSA, obstructive sleep apnea; IQR, interquartile range. Bold font indicates statistical significance

**Table 2:** Initial laboratory tests, SOFA score, and medications administered during the intubation period.

	Failed Weaning Group (N = 48) ^1^	Successful Weaning Group (N = 40) ^1^	*p*-Value ^2^
SOFA score			**0.011**
Mean [SD]	8.31 [1.47]	7.41 [1.36]	
Median (IQR)	8.00 (7.00, 9.00)	7.50 (6.00, 8.00)	
P/F ratio	89 (65, 145)	142 (67, 250)	0.052
pCO_2_	48 (38, 57)	47 (40, 55)	0.7
White blood cells (*10^3^/uL)	10.5 (8.7, 12.3)	11.5 (9.6, 13.9)	0.3
Hemoglobin (mg/dL)	9.30 (8.15, 10.68)	9.75 (8.90, 11.20)	0.086
Platelets (*10^3^/uL)	282 (217, 372)	319 (227, 412)	0.2
Glucose (mg/dL)	123 (102, 191)	133 (117, 165)	0.5
Urea (mg/dL)	74 (49, 109)	43 (33, 82)	**0.008**
Total bilirubin (mg/dL)	0.56 (0.43, 0.74)	0.47 (0.38, 0.60)	**0.047**
Creatinine (mg/dL)	0.85 (0.55, 1.40)	0.74 (0.43, 1.22)	0.12
Albumin (g/dL)	2.40 (2.15, 2.85)	2.70 (2.50, 3.20)	**0.002**
INR	1.17 (1.10, 1.39)	1.12 (1.07, 1.15)	0.066
Duration of diuretic (days)	13 (5, 26)	22 (13, 32)	0.078
Diuretic agents daily dose (total amount/duration [mg/day])	52 (37, 84)	38 (33, 53)	0.089
BMI (kg/m^2^)	29(25,37)	27(23,34)	0.16
Vasoactive agents	17 (35%)	10 (25%)	0.3
Atracurium (once)	2 (4.2%)	1 (2.5%)	>0.9
Atracurium (twice or more)	0 (0%)	0 (0%)	
Steroids (at least once)	22 (46%)	22 (55%)	0.4

^1^ Median (IQR); ^2^ Wilcoxon rank-sum test; SOFA score: Sequential Organ Failure Assessment score, P/F ratio = PaO_2_ (mmHg) / FiO_2_, fraction of inspired oxygen, INR: international ratio, IQR: interquartile range; WBC: white blood cell. Bold font indicates statistical significance.

**Table 3:** Outcome data.

Characteristic	Overall, N = 88 ^1^	Failed Weaning Group, N = 48 ^1^	Successful Weaning Group, N = 40 ^1^	*p*-Value ^2^
Hospitalization length of stay (days)	45 (32, 59)	39 (31, 56)	52 (32, 61)	0.10
Duration of mechanical ventilation (days)	31 (19, 41)	35 (25, 48)	28 (18, 37)	0.040
In-hospital tracheostomy	69 (78%)	38 (79%)	31 (78%)	0.8
ICU days	15 (9, 25), n = 59	13 (9, 23), n = 32	16 (10, 26), n = 27	0.4
Mortality during hospitalization	25 (28%)	25 (52%)	0 (0%)	<0.001
All-cause mortality within 30 days since hospital discharge	10 (11%)	3 (6.3%)	7 (18%)	0.2
All-cause mortality within 1 year since hospital discharge	25 (28%)	12 (25%)	13 (33%)	0.4
Status at discharge				<0.001
Mechanical ventilation	23 (37%)	23 (100%)	0 (0%)	
Non-support	23 (37%)	0 (0%)	23 (58%)	
Oxygen	15 (24%)	0 (0%)	15 (38%)	
Oxygen + BiPAP	2 (3.2%)	0 (0%)	2 (5.0%)	

^1^ Median (IQR); n (%); Median (IQR), n = N; ^2^ Wilcoxon rank-sum exact test; Pearson’s Chi-square test; Wilcoxon rank-sum test; Fisher’s exact test ICU: intensive care unit, IQR: interquartile range.

**Table 4:** Logistic regression for predicting successful weaning from mechanical ventilation.

Characteristic	OR	95% CI	*p*-Value
SOFA score	0.54	0.32, 0.85	0.012
Albumin	5.71	1.79, 21.4	0.005
Age	0.97	0.93, 1.01	0.2
Urea	1.01	0.99, 1.02	0.3

OR: odds ratio, CI: confidence interval, SOFA score: Sequential Organ Failure Assessment score.

## Data Availability

No new data were created or analyzed in this study. The original contributions presented in this study are included in the article. Further inquiries can be directed to the corresponding author.

## Supplementary Materials

The following supporting information can be downloaded at: https://www.mdpi.com/article/10.3390/jcm14134427/s1, Table S1: Main reasons for intubation; Table S2: Logistic regression analysis for predicting successful weaning from mechanical ventilation.

## Abbreviations

The following abbreviations are used in this manuscript:

BiPAP	Bilevel Positive Airway Pressure
CKD	Chronic Kidney Disease
COPD	Chronic Obstructive Pulmonary Disease
FW	Failed Weaning
ICU	Intensive Care Unit
IQR	Interquartile Range
MV	Mechanical Ventilation
OSA	Obstructive Sleep Apnea
P/F Ratio	Partial Pressure of Oxygen (PaO_2_)/ Fraction of Inspired Oxygen (FiO_2_) Ratio
SBT	Spontaneous Breathing Trial
SUMC	Soroka University Medical Center
SW	Successful Weaning
WIND	Weaning Outcome According to a New Definition

## References

[1] McConville J.F., Kress J.P. (2012). Weaning patients from the ventilator. N. Engl. J. Med..

[2] Boles J.M., Bion J., Connors A., Herridge M., Marsh B., Melot C., Pearl R., Silverman H., Stanchina M., Vieillard-Baron A. (2007). Weaning from mechanical ventilation. Eur. Respir. J..

[3] Pilcher D.V., Bailey M.J., Treacher D.F., Hamid S., Williams A.J., Davidson A.C. (2005). Outcomes, cost and long term survival of patients referred to a regional weaning centre. Thorax.

[4] Epstein S.K., Ciubotaru R.L. (1998). Independent effects of etiology of failure and time to reintubation on outcome for patients failing extubation. Am. J. Respir. Crit. Care Med..

[5] Thille A.W., Richard J.C.M., Brochard L. (2013). The decision to extubate in the intensive care unit. Am. J. Respir. Crit. Care Med..

[6] Peñuelas O., Frutos-Vivar F., Fernández C., Anzueto A., Epstein S.K., Apezteguía C., González M., Nin N., Raymondos K., Tomicic V. (2011). Characteristics and outcomes of ventilated patients according to time to liberation from mechanical ventilation. Am. J. Respir. Crit. Care Med..

[7] Tonnelier A., Tonnelier J.M., Nowak E., Gut-Gobert C., Prat G., Renault A., Boles J.M., L’Her E. (2011). Clinical relevance of classification according to weaning difficulty. Respir. Care.

[8] Funk G.C., Anders S., Breyer M.K., Burghuber O.C., Edelmann G., Heindl W., Hinterholzer G., Kohansal R., Schuster R., Hartl S. (2010). Incidence and outcome of weaning from mechanical ventilation according to new categories. Eur. Respir. J..

[9] Pu L., Zhu B., Jiang L., Du B., Zhu X., Li A., Li G., He Z., Chen W., Ma P. (2015). Weaning critically ill patients from mechanical ventilation: A prospective cohort study. J. Crit. Care.

[10] Jeong B.H., Ko M.G., Nam J., Yoo H., Chung C.R., Suh G.Y., Jeon K. (2015). Differences in clinical outcomes according to weaning classifications in medical intensive care units. PLoS ONE.

[11] Schönhofer B., Euteneuer S., Nava S., Suchi S., Köhler D. (2002). Survival of mechanically ventilated patients admitted to a specialised weaning centre. Intensive Care Med..

[12] Lago A.F., Gastaldi A.C., Mazzoni A.A.S., Tanaka V.B., Siansi V.C., Reis I.S., Basile-Filho A. (2019). Comparison of International Consensus Conference guidelines and WIND classification for weaning from mechanical ventilation in Brazilian critically ill patients: A retrospective cohort study. Medicine.

[13] Sellares J., Ferrer M., Cano E., Loureiro H., Valencia M., Torres A. (2011). Predictors of prolonged weaning and survival during ventilator weaning in a respiratory ICU. Intensive Care Med..

[14] Upadya A., Tilluckdharry L., Muralidharan V., Amoateng-Adjepong Y., Manthous C.A. (2005). Fluid balance and weaning outcomes. Intensive Care Med..

[15] Wiedemann H.P., Wheeler A.P., Bernard G.R., Thompson B.T., Hayden D., deBoisblanc B., Connors A.F., Hite R.D., Harabin A.L., National Heart, Lung, and Blood Institute Acute Respiratory Distress Syndrome (ARDS) Clinical Trials Network (2006). Comparison of two fluid-management strategies in acute lung injury. N. Engl. J. Med..

[16] Hjortrup P.B., Haase N., Bundgaard H., Thomsen S.L., Winding R., Pettilä V., Aaen A., Lodahl D., Berthelsen R.E., Christensen H. (2016). Restricting volumes of resuscitation fluid in adults with septic shock after initial management: The Classic randomised, parallel-group, multicentre feasibility trial. Intensive Care Med..

[17] Cinotti R., Lascarrou J.B., Azais M.A., Colin G., Quenot J.P., Mahé P.J., Roquilly A., Gaultier A., Asehnoune K., Reignier J. (2021). Diuretics decrease fluid balance in patients on invasive mechanical ventilation: The randomized-controlled single blind, IRIHS study. Crit. Care.

[18] Virole S., Duceau B., Morawiec E., Nierat M.C., Paifait M., Decaveie M., Demoule A., Delemazure J., Dres M. (2024). Contribution and evolution of respiratory muscles function in weaning outcome of ventilator-dependent patients. Crit. Care.

[19] Pham T., Heunks L., Bellani G., Madotto F., Aragao I., Beduneau G., Goligher E.C., Grasselli G., Laake J.H., Mancebo J. (2023). Weaning from mechanical ventilation in intensive care units across 50 countries (WEAN-SAFE): A multicentre, prospective, observational cohort study. Lancet Respir. Med..

[20] Béduneau G., Pham T., Schortgen F., Piquilloud L., Zogheib E., Jonas M., Grelon F., Runge I., Terzi N., Grangé S. (2017). Epidemiology of Weaning Outcome According to a New Definition. The WIND study. Am. J. Respir. Crit. Care Med..

[21] Windisch W., Dellweg D., Geiseler J., Westhoff M., Pfeifer M., Suchi S., Schönhofer B. (2020). Prolonged weaning from mechanical ventilation. Dtsch Ärztebl Int..

[22] Warnke C., Heine A., Müller-Heinrich A., Knaak C., Friesecke S., Obst A., Bollmann T., Desole S., Boesche M., Stubbe B. (2020). Predictors of survival after prolonged weaning from mechanical ventilation. J. Crit. Care.

[23] Saber H., Palla M., Kazemlou S., Navi B.B., Yoo A.J., Simonsen C.Z., Sandio A., Rajah G., Khatibi K., Liebeskind D.S. (2021). Prevalence, predictors, and outcomes of prolonged mechanical ventilation after endovascular stroke therapy. Neurocrit. Care.

[24] Magnet F.S., Bleichroth H., Huttmann S.E., Callegari J., Schwarz S.B., Schmoor C., Windisch W., Storre J.H. (2018). Clinical evidence for respiratory insufficiency type II predicts weaning failure in long-term ventilated, tracheotomised patients: A retrospective analysis. J. Intensive Care..

[25] Ghauri S.K., Javaeed A., Mustafa K.J., Khan A.S. (2019). Predictors of prolonged mechanical ventilation in patients admitted to intensive care units: A systematic review. Int. J. Health Sci. (Qassim).

[26] Huang M., Ong B.H., Hoo A.E.E., Gao F., Chao V.T.T., Lim C.H., Tan T.E., Sin K.Y.K. (2020). Prognostic factors for survival after extracorporeal membrane oxygenation for cardiogenic shock. ASAIO J..

[27] Wiedermann C.J. (2021). Hypoalbuminemia as surrogate and culprit of infections. Int. J. Mol. Sci..

[28] Conde M., Lawrence V. (2008). Postoperative pulmonary infections. BMJ Clin. Evid..

[29] Moodie L., Reeve J., Elkins M. (2011). Inspiratory muscle training increases inspiratory muscle strength in patients weaning from mechanical ventilation: A systematic review. J. Physiother..

