# Supplementary material for: Predictors of Weaning Success in Patients on Prolonged Mechanical Ventilation: A Retrospective Cohort Study
Source: J Clin Med. 2025 Jun 22;14(13):4427. doi: 10.3390/jcm14134427 (PMC12249621; doi:10.3390/jcm14134427)
Supplement: Supplementary file 1 [file jcm-14-04427-s001.zip › jcm-3663868-supplementary.pdf]

Table S1. Main reasons for intubation.

Characteristic	Overall, N = 88[1]	Failed weaning group, N = 48[1]	Successful weaning group, N = 40[1]	P-value[2]
Intubation main reason				0.036
Other	16 (18%)	9 (19%)	7 (18%)	
Sepsis	15 (17%)	9 (19%)	6 (15%)	
Pneumonia	14 (16%)	6 (13%)	8 (20%)	
Respiratory failure	9 (10%)	9 (19%)	0 (0%)	
CVA	7 (8.0%)	2 (4.2%)	5 (13%)	
Myocardial infarction	6 (6.8%)	4 (8.3%)	2 (5.0%)	
Traumatic brain injury	6 (6.8%)	3 (6.3%)	3 (7.5%)	
COPD exacerbation	5 (5.7%)	1 (2.1%)	4 (10%)	
Cardiac arrest	4 (4.5%)	3 (6.3%)	1 (2.5%)	
COVID-19	3 (3.4%)	2 (4.2%)	1 (2.5%)	
Suicide	3 (3.4%)	0 (0%)	3 (7.5%)	

[1] n (%)

[2] Fisher's exact test

CVA, cerebrovascular accident; COPD, chronic obstructive pulmonary disease; COVID-19, coronavirus disease 2019

Table S2. Logistic regression analysis for predicting successful weaning from mechanical ventilation.

Characteristic	OR[1]	95% CI[1]	*p*-value
Albumin	7.27	2.00, 34.6	0.006
Diuretic daily dose	0.98	0.96, 1.00	0.062
Urea	1.00	0.99, 1.01	0.8
First P/F ratio	1.00	1.00, 1.01	0.14

[1] OR: odds ratio, CI: confidence interval
